# Atomically Dispersed Iron Active Sites Promoting Reversible Redox Kinetics and Suppressing Shuttle Effect in Aluminum–Sulfur Batteries

**DOI:** 10.1007/s40820-022-00915-4

**Published:** 2022-08-20

**Authors:** Fei Wang, Min Jiang, Tianshuo Zhao, Pengyu Meng, Jianmin Ren, Zhaohui Yang, Jiao Zhang, Chaopeng Fu, Baode Sun

**Affiliations:** School of Materials Science and Engineering, Shanghai Jiao Tong University, Shanghai, 200240 People’s Republic of China

**Keywords:** Fe single atom, Aluminum–sulfur battery, Catalysis, Shuttle effect, Separator modification

## Abstract

Rechargeable aluminum–sulfur (Al–S) batteries have been considered as a highly potential energy storage system owing to the high theoretical capacity, good safety, abundant natural reserves, and low cost of Al and S. However, the research progress of Al–S batteries is limited by the slow kinetics and shuttle effect of soluble polysulfides intermediates. Herein, an interconnected free-standing interlayer of iron single atoms supported on porous nitrogen-doped carbon nanofibers (FeSAs-NCF) on the separator is developed and used as both catalyst and chemical barrier for Al–S batteries. The atomically dispersed iron active sites (Fe–N_4_) are clearly identified by aberration-corrected high-angle annular dark-field scanning transmission electron microscopy and X-ray absorption near-edge structure. The Al–S battery with the FeSAs-NCF shows an improved specific capacity of 780 mAh g^−1^ and enhanced cycle stability. As evidenced by experimental and theoretical results, the atomically dispersed iron active centers on the separator can chemically adsorb the polysulfides and accelerate reaction kinetics to inhibit the shuttle effect and promote the reversible conversion between aluminum polysulfides, thus improving the electrochemical performance of the Al–S battery. This work provides a new way that can not only promote the conversion of aluminum sulfides but also suppress the shuttle effect in Al–S batteries.

**Supplementary Information:**

The online version contains supplementary material available at 10.1007/s40820-022-00915-4.

## Highlights

- Fe single atoms supported on porous carbon nanofiber are prepared by spatial confinement.
- The iron single atoms supported on porous nitrogen-doped carbon nanofibers (FeSAs-NCF) can promote the reversible conversion between aluminum polysulfides.
- The FeSAs-NCF can chemically anchor the polysulfides to suppress shuttle effect.

**Supplementary Information:**

The online version contains supplementary material available at 10.1007/s40820-022-00915-4.

## Graphical abstract

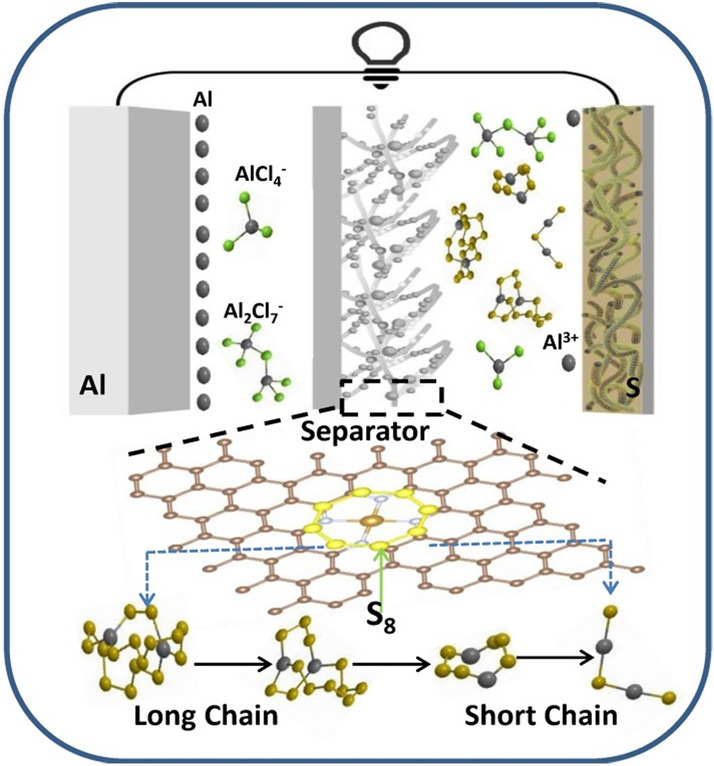

## Introduction

Metal–sulfur batteries, using sulfur as a cathode material coupled with metal anodes, have become one of the most highly potential energy storage systems due to the high energy density, rich raw materials, low cost and environmental-friendliness [1]. Currently, various metal–sulfur batteries have been reported, and lithium–sulfur batteries are the most extensively studied. Although lithium–sulfur battery can offer a high theoretical capacity, the high cost of lithium anode arose from the finite lithium resource and the safety issue arose from lithium dendrite formation hindering the practical application [2–4]. Aluminum as the most abundant metal element in the crust can couple with sulfur to fabricate rechargeable aluminum–sulfur (Al–S) batteries, in which both aluminum and sulfur are cheap and environmentally friendly materials with good air stability and safety. More importantly, both Al and S electrodes have high theoretical specific capacities of 2976 and 1672 mAh g^−1^, respectively [5–7]. A rechargeable Al–S battery can deliver a theoretical cell capacity of 1072 mAh g^−1^ based on total electrode mass and operate at a voltage of 1.25 V, resulting in a theoretical energy density of 1340 Wh kg^−1^ [8]. Therefore, Al–S batteries are promising for large-scale energy storage.

Rechargeable Al–S battery was first proposed in 2015, and a high specific capacity was delivered; however it could only run a few cycles [9]. Since then, various electrode materials and electrolytes were explored to boost electrochemical performances of Al–S batteries [8, 10–19]. Currently, the main problems of Al–S batteries are slow kinetic response and short cycle life. The slow kinetics is mainly caused by the charge transfer reaction between sulfur and Al and the dissociation barrier of the aluminum species [6, 11, 20]. The short cycle life is caused by the following reasons: (1) the aluminum polysulfides intermediate products can dissolve in the electrolyte and shuttle to the Al anode, leading to the loss of active sulfur; (2) the slow kinetic response of reversible conversion between Al_2_S_3_ and S causes gradual sulfur loss, further leading to poor life-span; (3) the strong bond energy of Al–S may cause incomplete reaction of Al_2_S_3_, which also shortens the cycle life [8, 9, 21]. At present, different strategies have been proposed to address the above problems. One is using other halogen anions to replace chloride ions in electrolytes to lower the dissociation barrier of aluminum ion clusters. For example, the brominated electrolyte could increase the reaction kinetics, because the energy barrier of releasing Al^3+^ was lower than that of Al_2_Cl_7_^−^ [11]. The second is introducing catalysts to promote the conversion of aluminum polysulfides during charge/discharge processes. Wan and co-workers fabricated a Cu/Co-porous carbon composite, in which Cu and Co species as active centers promoted the conversion of aluminum polysulfides during the charge–discharge processes. Specifically, the Cu formed an ionic cluster with polysulfide to facilitate the reversibility of S, while the Co contributed to form cobalt sulfides, and the assembled Al–S battery showed an enhanced cycle performance [22, 23]. The third is adding chemical or physical barriers to suppress the dissolution of aluminum polysulfides [24]. The strong interactions between lithium polysulfides and heteroatoms doped carbon or polymeric carbon nitride have been discussed and confirmed in order to anchor polysulfides [25–27]. Stimulated by these research approaches of covalent binding and ionic association in lithium–sulfur batteries [28–30], similar approaches can also be learned to effectively capture polysulfides and inhibit shuttle effect for Al–S batteries. However, it is difficult to solve the issues of slow kinetics and short cycle life concurrently through the above approaches. Therefore, it is necessary to find a way that can not only reduce the dissociation barrier of aluminum ion clusters and promote the conversion of aluminum sulfides but also suppress the shuttle effect, simultaneously.

Recently, isolated single atoms supported on carbon matrix have been regarded as efficient catalysts for electrocatalysis (e.g., oxygen reduction reaction) due to the unique structure, highly dispersed active sites and the maximum atomic utilization [31, 32]. Organic metallic materials with isolated metal sites are also promising for batteries, and the organic aluminum compound with Al–O coordination can modulate the deposition of lithium [33]. Single atom catalysts (SACs) were employed in lithium–sulfur batteries for enhancing electrochemical performance through the strong interaction between single atoms and lithium polysulfides [34, 35]. Inspired by this, it is expected that an interconnected free-standing interlayer composed of SACs with distinctive electronic structure on the separator may not only accelerate the charge transfer between aluminum and sulfur but also chemically interact with aluminum polysulfides and physically block soluble aluminum polysulfides to suppress the shuttle effect [32].

Herein, iron single atoms supported on porous nitrogen-doped carbon nanofibers (FeSAs-NCF) with free-standing network are fabricated and directly coated onto the separator to form the interlayer of Al–S batteries. The iron single atoms are clearly identified by HAADF-STEM and XANES. The Al–S battery with the FeSAs-NCF shows an improved specific capacity and enhanced cycle stability, as the specific capacity remains 320 mAh g^−1^ at a current density of 1000 mA g^−1^ after 500 cycles. The improved electrochemical performance is explained that the atomically dispersed iron active centers can promote the reversible conversion between aluminum polysulfides to accelerate reaction kinetics, stabilize aluminum anode, chemically anchor the polysulfides and physically block soluble aluminum polysulfides to suppress the shuttle effect, which are evidenced by the experimental and theoretical results.

## Experimental and Calculation

### Synthesis of FeSAs-NCF

The precursor of FeSAs-CNF was first prepared (see Supporting Information). The obtained nanofibers were pre-oxidized at 250 °C with a heating rate of 1 °C min^−1^ in air for 2 h. The samples were then carbonized by utilizing a plasma enhanced chemical vapor deposition (PECVD) system. The obtained samples were treated by N_2_ plasma (plasma power of 300 W) at 500 °C (2 °C min^−1^) for 5 min a pressure of 12.9 Pa and then calcinated at 920 °C (2 °C min^−1^) for 2 h under N_2_ atmosphere.

### Preparation of S Cathodes

First, the composite of S and carbon nanofibers (S@CNF) was obtained by heating the mixture of the both at 155 °C for 12 h, and the content of sulfur is 69%. The as-prepared S@CNF was mixed with carbon black and polyvinylidene fluoride (PVDF), in methylpyrrolidone (NMP). Then, the S@CNF slurry was casted onto Mo foil, which was used as the current collector due to the high corrosion resistance to ionic liquids.

### Modification of Separator

The sheet shape of FeSAs-NCF (Fig. S1) was directly coated onto the Whatman GF/D glass fiber separator and then punched into the disks with a diameter of 14 mm.

### Electrochemical Measurements

The Al–S batteries were assembled in Swagelok-type cells. Al foil was used as the anode, the separator was Whatman GF/D, and the electrolyte was [EMIM]Cl/AlCl_3_ (mole ratio: 1:1.3); the cells were assembled in glovebox (O_2_, H_2_O < 1 ppm). Galvanostatic charge/discharge was performed within the voltage range between 0.01 and 1.8 V. The cells were measured using a LAND battery system under different current densities. Before long-term cycling, the assembled battery firstly was run for two cycles. Electrochemical impedance spectroscopy (EIS) was performed within the frequency range from 1 MHz to 0.01 Hz (Gamry REF 600).

### Calculation

All theoretical calculations were carried out in the Vienna *ab*-initio simulation package (VASP) using projector augmented wave (PAW) method to gain the system energy and structural information. The generalized gradient approximation (GGA) with the Perdew–Burke–Ernzerhof (PBE) functional was adopted in all calculations. A supercell of graphene containing 6 × 6 × 1 unit cells was used in all graphene-based systems. In the vertical direction, a vacuum layer of about 15 Å in thickness was introduced in the surfaces for all composite systems (polysulfide and graphene-based systems). The cutoff energy was set at 520 eV, and total energy convergence was set to be lower than 1 × 10^−5^ eV. The first Brillouin zone was sampled with a k-points mesh of 3 × 3 × 3 Gamma-centered grids for the structural relaxation.

## Results and Discussion

**Fig. 1:**
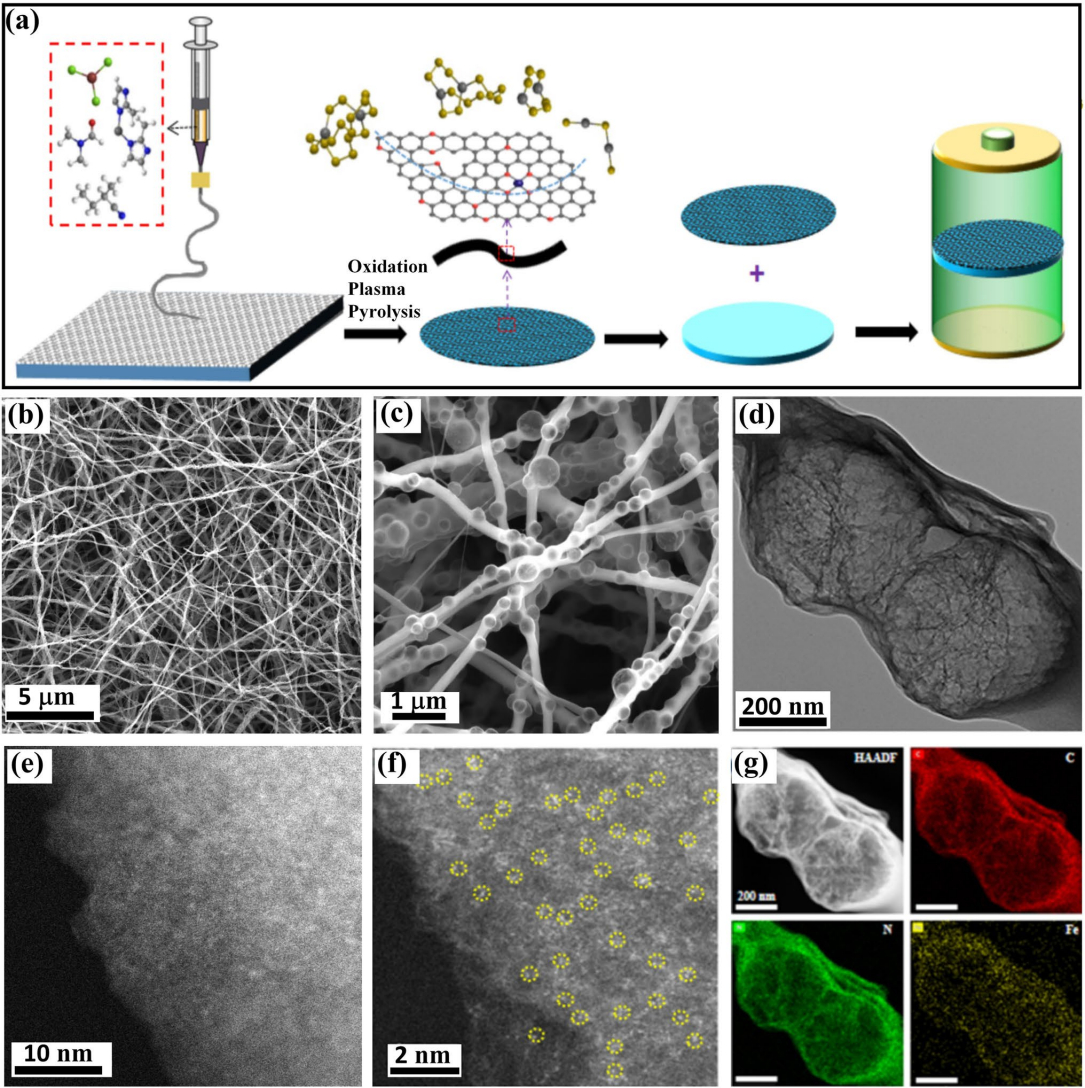
**a** Synthetic illustration of the FeSAs-NCF. **b, c** SEM, **d** TEM, **e, f** aberration-corrected HAADF-STEM images and **g** the corresponding element mappings of the FeSAs-NCF

The synthesis of the FeSAs-NCF is shown schematically in Fig. 1a. First, polyacrylonitrile (PAN), iron phenanthroline (Fe-Phen), and zinc oxide nanoparticles were dispersed in dimethylformamide and electrospun to obtain a piece of PAN-based cloth, and then it was pyrolyzed with the aid of N_2_ plasma. During the plasma-assisted pyrolysis, the PAN was converted to porous nitrogen-doped carbon nanofibers, and all the Fe-Phen was converted to Fe single atoms coordinated with nitrogen atoms. Meanwhile, the zinc oxide nanoparticles were reduced and volatilized to leave a large number of pores in the nanofibers. As a result, a sheet of FeSAs-NCF was successfully obtained (Fig. S1) and FeSAs-NCF coating on the separator can form the physical barrier to alleviate the shuttle effect. To further analyze the FeSAs-NCF, it displays nanofiber structure and there are abundant pores inside the nanofibers (Fig. 1b-c) through the scanning electron microscopy images (SEM). The one-dimensional porous nanofiber structure can facilitate to expose more active sites, shorten ion diffusion path and accelerate electron transfer to enhance electrochemical performance. The porous structure with a pore size of approximately 250 nm is further confirmed by the representative transmission electron microscopy (TEM) image in Fig. 1d. Additionally, there are no particles observed in the SEM or TEM images, suggesting the possible formation of Fe single atoms. The corresponding selected area electron diffraction (SAED) also suggests the absence of Fe-related crystal phase (Fig. S2), further confirming the formation of Fe single atoms. HAADF-STEM images display the homogeneously distributed Fe single atoms (Fig. 1e-f), which are clearly identified throughout the carbon nanofibers in the enlarged image (marked in yellow circles in Fig. 1f). Additionally, the elemental mappings of the FeSAs-NCF evidence the homogeneous distribution of Fe, C and N in the nanofibers (Fig. 1g). The mass loading of iron single atoms was determined to be 2.94 wt% by inductively coupled plasma-mass spectrometry. These a large number of iron atoms with highly dispersed state can maximize atomic utilization and act as active sites to improve the electrochemical performance.

**Fig. 2:**
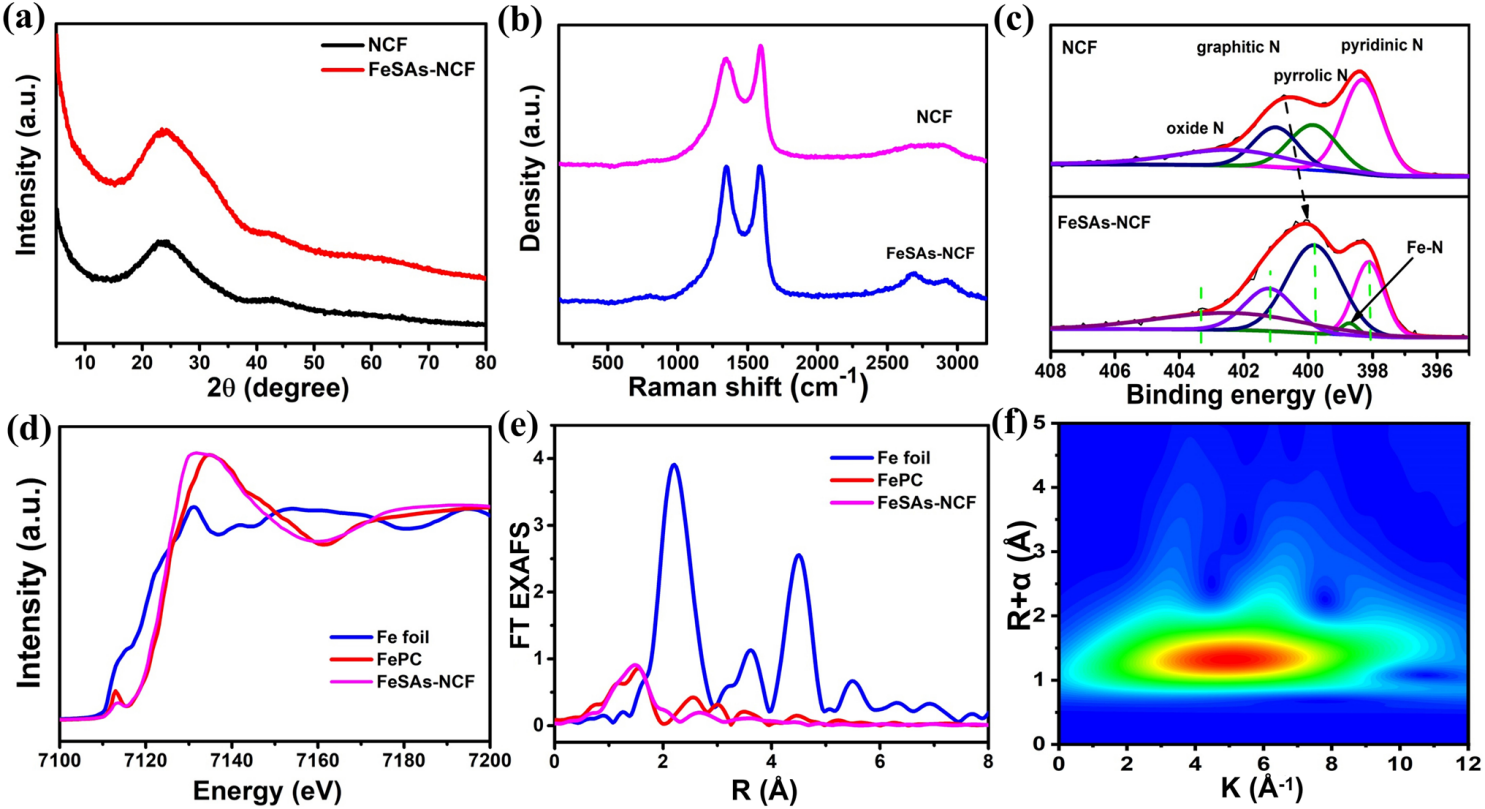
**a** XRD patterns, **b** Raman spectra and **c** N 1*s* XPS of the FeSAs-NCF and NCF. **d** XANES and **e** FT EXAFS spectra of the FeSAs-NCF, Fe foil and FePc. **f** Wavelet transforms of the FeSAs-NCF

The FeSAs-NCF displays the identical XRD pattern of the NCF, and the two diffraction peaks of graphitic carbon are assigned to the (002) and (101) crystal planes (Fig. 2a) [36]. The absence of any Fe-related phase confirms the atomic dispersion of Fe in the N-doped carbon nanofibers, which is in good accordance with the STEM observation. The specific surface area of the FeSAs-NCF is 227.8 m^2^ g^−1^ estimated from nitrogen adsorption–desorption isotherm (Fig. S3), and the mesopores with the main pore size of ~ 30 nm mainly exist in the FeSAs-NCF (Fig. S4). Furthermore, the FeSAs-NCF displays a larger *I*_D_/*I*_G_ value (1.00) than that of the NCF (0.91), demonstrating that the defective degree is enhanced by introducing the iron single atoms (Fig. 2b). The result of X-ray photoelectron spectroscopy (XPS) could confirm iron, nitrogen, and carbon elements in the FeSAs-NCF (Fig. S5). The N 1*s* XPS of the NCF is divided into four component peaks at 398.1, 399.8, 401.2, and 402.4 eV, which are assigned to pyridinic N, pyrrolic N, graphitic N, and oxidized N, respectively (Fig. 2c) [37, 38]. Compared with the NCF, an additional peak of N 1*s* of the FeSAs-NCF appears at 398.8 eV, which is identified as Fe–N bond [37, 38]. XANES was used to further identify the electronic structure and coordination of the FeSAs-NCF. Figure 2d shows the Fe K-edge XANES spectra of the FeSAs-NCF, Fe foil, and FePc. The near-edge absorption energy of Fe in the FeSAs-NCF is close to that of FePc but totally different from that of Fe foil, demonstrating that the Fe is atomically dispersed and coordinated with N as Fe–N_4_ [39, 40]. The small peak at ~ 7113.5 eV is associated with the transition from 1*s* to 4*pz*, implying the existence of Fe–N_4_ configuration [41, 42]. The Fourier transformed (FT) k^2^-weighted-EXAFS spectra of FeSAs-NCF, Fe foil, and FePc are displayed in Fig. 2e, and the main peak at 1.6 Å of the FeSAs-NCF is attributed to the Fe–N coordination [41, 42]. The obtained Fourier transformed (FT) k^2^-weighted-EXAFS spectrum was fitted to identify the coordination number of Fe in the FeSAs-NCF. The calculated coordination number of Fe–N is ~ 3.52 in the first shell of Fe atoms for FeSAs-NCF (Fig. S6 and Table S1). The wavelet transform-EXAFS (WT-EXAFS) of Fe k-edge was performed to further distinguish the dispersion of Fe in the FeSAs-NCF. The WT contour plot of the FeSAs-NCF only shows the maximum intensity peak at 5 Å^−1^ (Fig. 2f), and this maximum intensity peak is similar to that of FePc but different from that of Fe foil (Fig. S7), further confirming the Fe–N coordination. The results clearly evidence the Fe sites are atomically dispersed with the coordination of Fe–N_4_ in the FeSAs-NCF.

**Fig. 3:**
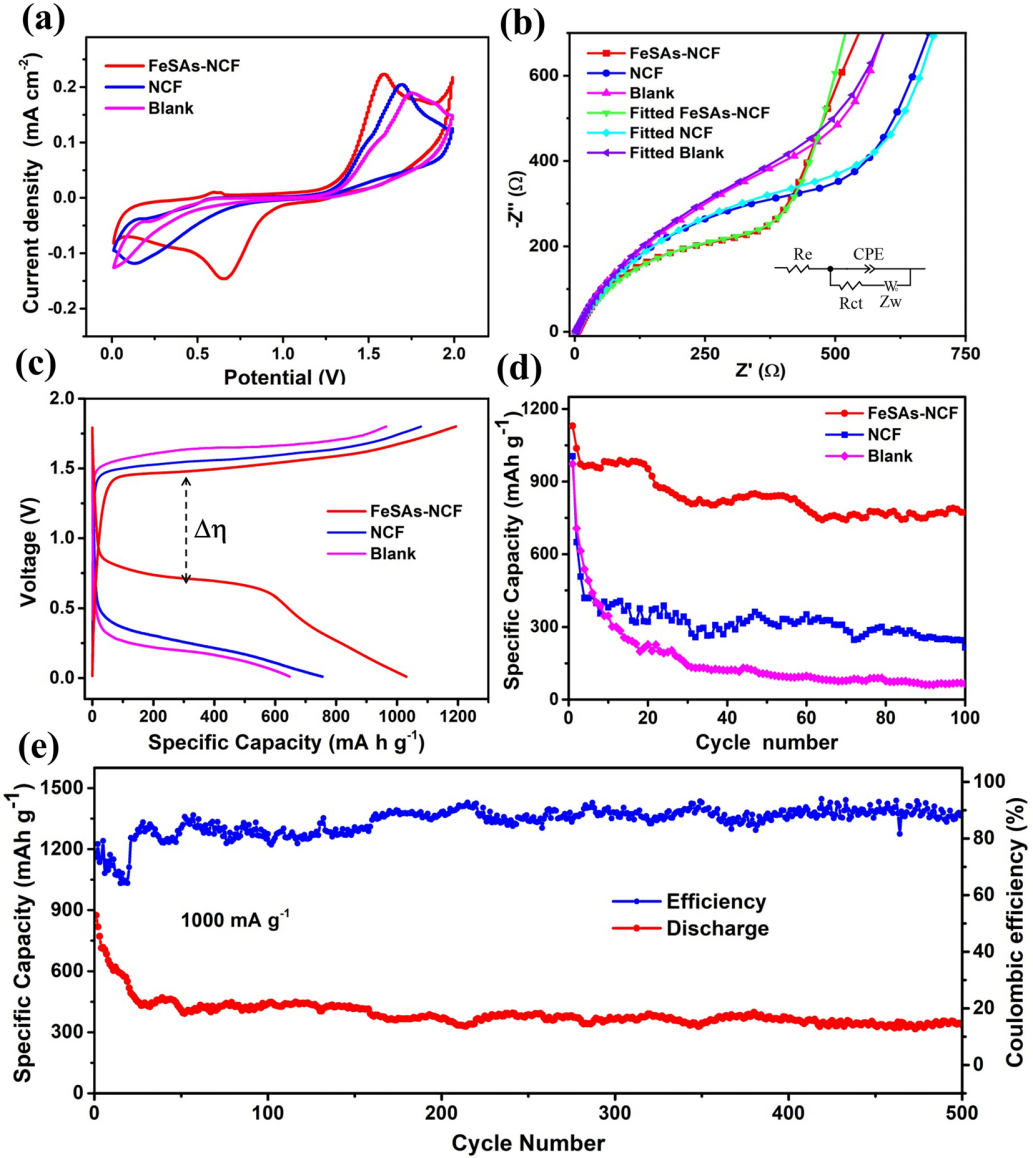
**a** CV curves, **b** Nyquist plots, **c** charge–discharge curves and **d** cycling stability of the Al–S batteries with FeSAs-NCF and NCF, and the blank cell, **e** cycling stability of the cell with FeSAs-NCF at 1000 mA g.^−1^

Al–S batteries with various modified separators were assembled and evaluated to verify the catalysis and anchor roles of FeSAs-NCF. The thickness of the FeSAs-NCF layer on the separator is ~ 30 μm (Fig. S8). The representative cyclic voltammetry (CV) curves clearly demonstrate the reversible redox reactions between aluminum and sulfur in the Al–S batteries (Fig. 3a) [23]. Meanwhile, the Al–S battery with the FeSAs-NCF displays larger redox peak current densities and a smaller peak potential separation than the cell with NCF and the blank cell, demonstrating the FeSAs-NCF can facilitate the redox reaction between Al and S and accelerate the redox kinetics of converting sulfur into aluminum sulfides. Additionally, the larger peak current densities of the Al–S battery with FeSAs-NCF indicate a higher conversion efficiency between sulfur and aluminum sulfides, which can boost the utilization of S and retard the shuttle effect of soluble aluminum polysulfides. The role of FeSAs-NCF in promoting charge transfer capability was evaluated by electrochemical impedance spectroscopy (EIS). The fitted Nyquist plots clearly indicate that the charge transfer resistance (*R*_ct_) of the Al–S battery with FeSAs-NCF is much smaller than that with the NCF and that of the blank one (Fig. 3b and Table S2), revealing the FeSAs-NCF can boost charge transfer capability of the Al–S battery. Then, the diffusion coefficients were calculated from the Nyquist plots in Fig. 3b to further evaluate reaction kinetics, and the diffusion coefficients of cells with FeSAs-NCF, NCF and blank separators are 9.63 × 10^–15^, 2.70 × 10^–15^, and 7.43 × 10^–16^ cm^2^ s^−1^ (see details in Supporting Information). Meanwhile, the ion diffusion coefficient was also studied by galvanostatic intermittent titration technique (Fig. S9), and the results display that the ratio of *D*_1_ (the cell with FeSAs-NCF) to *D*_2_ (the blank cell) is greater than 1, further revealing that the FeSAs-NCF can accelerate the ion diffusion to contribute to fast reaction kinetic.

The typical galvanostatic charge/discharge curves display that the cell with FeSAs-NCF shows a higher discharge voltage and delivers a larger discharge capacity than the cell with NCF and the blank cell (Fig. 3c). Furthermore, the cell with FeSAs-NCF displays the highest discharge voltage of 0.75 V, and this high discharge voltage plateau can still retain after 100 cycles (Fig. S10). Moreover, the cell with FeSAs-NCF obviously possesses a lower electrochemical polarization than the cell with NCF and blank cell, indicating the lowest energy barrier of the conversion reaction between charge and discharge products. Figure 3d shows that the cell with FeSAs-NCF delivers an initial capacity of 1130 mAh g^−1^ at 100 mA g^−1^ and remains a stable capacity of 780 mAh g^−1^ after 100 cycles. However, the battery with NCF and blank cell only remain the specific capacities of 280 and 70 mAh g^−1^ after 100 cycles, respectively. Furthermore, the battery with FeSAs-NCF demonstrates good cycling stability and the specific capacity reaches 320 mAh g^−1^ at a high current density of 1000 mA g^−1^ after 500 cycles (Fig. 3e). The Coulombic efficiency is approximately 90% upon cycling, and the possible reasons can be explained that: (1) the dissolution of discharge product polysulfides may not be completely suppressed, and there is still a small number of polysulfides migrating to the negative electrode side; (2) the discharge product Al_2_S_3_ is not fully oxidized due to the non-conducive nature. Additionally, the Al–S battery performances were compared with others reported in recent literature (Table S3), and the results display that combination of specific capacity and cycling stability in this work surpasses most of others. The enhanced electrochemical performance is explained that the FeSAs-NCF can accelerate reaction kinetics and chemically adsorb the polysulfides to promote the reversible conversion between aluminum polysulfides and inhibit the shuttle effect.

**Fig. 4:**
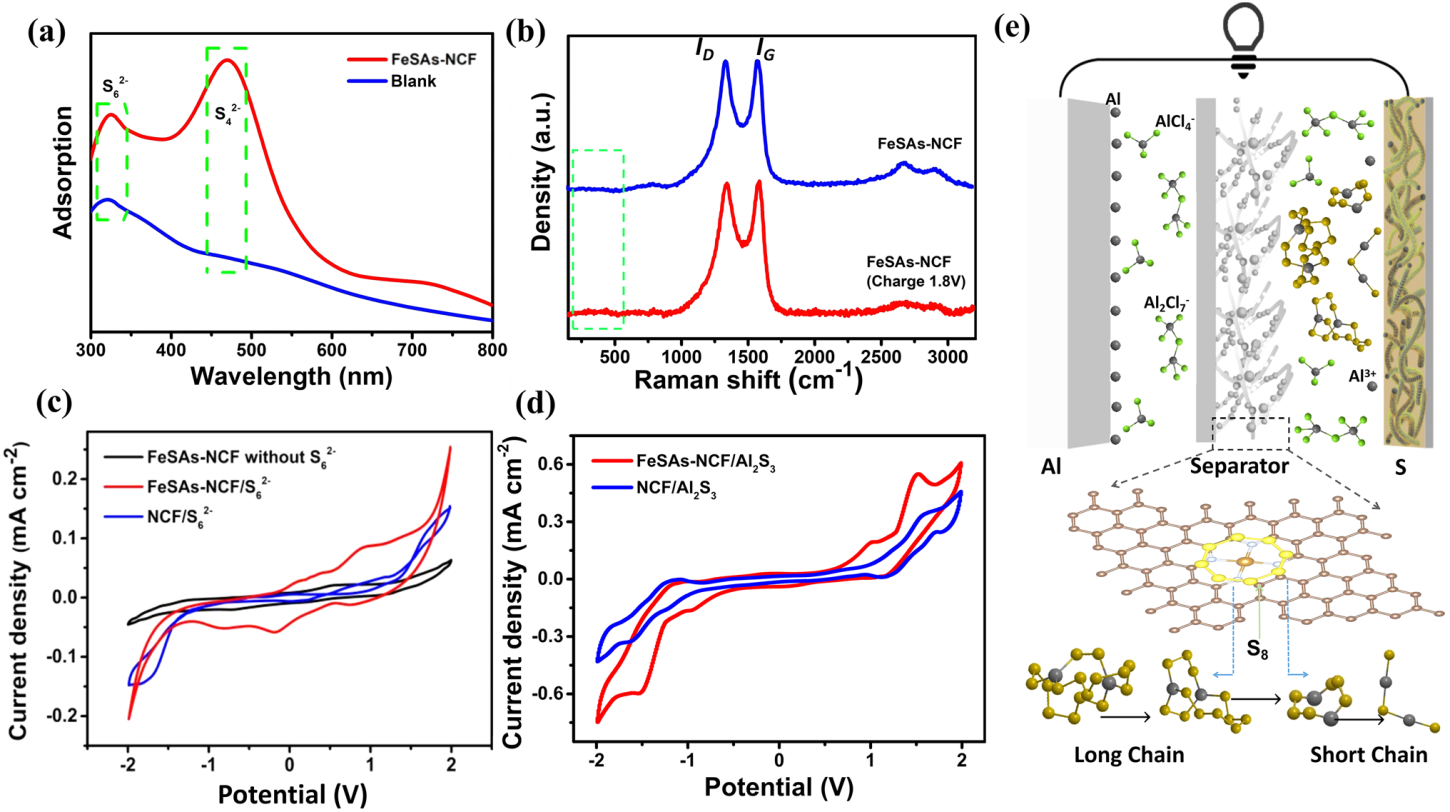
**a** UV–Vis spectra of the electrolytes in the Al–S batteries with FeSAs-NCF and the blank cell after charge/discharge. **b** Raman spectra of pristine FeSAs-NCF and the FeSAs-NCF collected at a charge state of 1.8 V. **c** CV curves of the symmetric cells in the electrolyte containing ${S}_{6}^{2-}$. **d** CV curves of the symmetric cells with Al_2_S_3_. **e** Schematic illustration of inhibiting shuttle effect and catalyzing aluminum polysulfides of the Al–S battery with FeSAs-NCF

The roles of FeSAs-NCF in catalyzing aluminum polysulfides and suppressing shuttle effect of the Al–S batteries were further investigated. Firstly, the morphologies of the anodes after cycling were analyzed. In the cells without FeSAs-NCF, the surfaces of the Al anodes after cycling are uneven and dark (Fig. S11), which is ascribed to the uneven dissolution and deposition of aluminum and the irreversible reactions of the soluble aluminum polysulfides caused by the shuttle effect. Meanwhile, in the Al–S cell with FeSAs-NCF, there is no obvious change of the Al anode surface after cycling, revealing that the shuttle effect is suppressed by the FeSAs-NCF, and the FeSAs interlayer contributes to the homogenization of aluminum deposition. A close observation displays that there are a large number of irregular holes in the Al anodes from the cell with NCF and the blank cell (Fig. S12), which is caused by the uneven dissolution and deposition of aluminum during charge–discharge process. The difference in the surface morphologies reveals that the FeSAs-NCF can induce well-distributed deposition of aluminum. The EDS mappings reveal that the sulfur-containing species were deposited on the Al anode without the addition of FeSAs-NCF (Fig. S13), inferring that the soluble aluminum polysulfides shuttled to the Al anode. To further verify the adsorption capacity of FeSAs-NCF on polysulfides, the permeation test was conducted for visualization using H-type cell. When the blank separator was used, the color of the solution in right chamber gradually gets darker (Fig. S14). However, there is no noticeable color change in right chamber after 48 h when the FeSAs-NCF was used, revealing the strong adsorption capacity of FeSAs-NCF on polysulfides. The results confirm that the strong barrier effect of the FeSAs-NCF to chemically anchor the soluble aluminum polysulfides. Subsequently, the electrolytes in Al–S batteries with and without FeSAs-NCF layer after cycling were collected and measured by ultraviolet spectrophotometer (UV–Vis). The UV–Vis spectra of the electrolytes in the cells with and without FeSAs-NCF collected at 1.8 V appear the same absorption peak at ~ 290 nm, which is assigned to the absorption peak of ${\mathrm{S}}_{6}^{2-}$(Fig. 4a) [22, 23, 43]. The difference is that the electrolyte in the cell with FeSAs-NCF shows another absorption peak at ~ 465 nm, corresponding to ${\mathrm{S}}_{4}^{2-}$ polysulfide in the electrolyte [22, 23, 43]. The high yield of ${\mathrm{S}}_{4}^{2-}$ polysulfide is possibly because the Fe–N_4_ active centers are more beneficial to catalyze ${\mathrm{S}}_{8}$ into ${\mathrm{S}}_{4}^{2-}$ than ${\mathrm{S}}_{8}$ into ${\mathrm{S}}_{6}^{2-}$ [22, 23, 43]. Additionally, compared with the pristine FeSAs-NCF, the Raman spectrum of the FeSAs-NCF collected at 1.8 V displays two additional light peaks within the range of 250–600 cm^−1^, which is ascribed to the chemically adsorbed polysulfides (${\mathrm{S}}_{\mathrm{n}}^{2-}$). The result clearly confirms the adsorption of polysulfides on the FeSAs-NCF [44]. Then, the aluminum polysulfide (${\mathrm{S}}_{6}^{2-}$) was prepared and employed to assemble symmetric cells to further evaluate the catalytic role of the FeSAs-NCF (Fig. S15). The CV curve of the FeSAs-NCF symmetric cell shows two obvious reduction peaks at −0.25 V and −0.90 V compared with that in the electrolyte without ${\mathrm{S}}_{6}^{2-}$, which are identified as the reduction of ${\mathrm{S}}_{6}^{2-}$ to Al_2_S_3_ (Fig. 4c). However, there are almost no such reduction peaks in the CV curve of the symmetric cell with NCF. The result further confirms that the FeSAs-NCF can catalytic the reduction of ${\mathrm{S}}_{6}^{2-}$. Another symmetric cell with FeSAs-NCF/Al_2_S_3_ as both anode and cathode was also studied. The corresponding CV curve displays that the peak current densities of the FeSAs-NCF/Al_2_S_3_ symmetric cell are much larger than that of the NCF/Al_2_S_3_ cell, indicating that the FeSAs-NCF can obviously accelerate the reaction rate of the Al_2_S_3_ (Fig. 4d). Moreover, compared with the NCF/Al_2_S_3_ symmetric cell, the FeSAs-NCF/Al_2_S_3_ symmetric cell shows the smallest voltage hysteresis between the oxidation and reduction peaks, confirming the atomically dispersed Fe active centers are more favorable to promote the kinetic conversion of Al_2_S_3_ (Fig. 4d). All the above results experimentally verify that the FeSAs-NCF can not only suppress the shuttle effect but also catalyze the redox reactions of aluminum polysulfides for the Al–S battery. Based on the above analysis and discussion, the mechanism of the FeSAs-NCF inhibiting shuttle effect and catalyzing aluminum polysulfides is illustrated in Fig. 4e, and the discharge reactions on cathode side is expressed as follows:

$$\begin{gathered} {\text{32Al}}_{{2}} {\text{Cl}}_{{7}}^{ - } + {\text{ 9S}}_{{8}} + {\text{4FeN}}_{{4}} + {\text{ 24e}}\mathop{\longrightarrow}\limits^{{}}{\text{4Al}}_{{2}} {\text{S}}_{{{18}}}^{*} - {\text{FeN}}_{{4}} + {\text{ 56AlCl}}_{{4}}^{ - } \hfill \\ {\text{8Al}}_{{2}} {\text{Cl}}_{{7}}^{ - } + {\text{ 2Al}}_{{2}} {\text{S}}_{{{18}}}^{*} - {\text{FeN}}_{{4}} + {\text{FeN}}_{{4}} + {\text{6e}}\mathop{\longrightarrow}\limits^{{}}{\text{3Al}}_{{2}} {\text{S}}_{{{12}}}^{*} - {\text{FeN}}_{{4}} + {\text{ 14AlCl}}_{{4}}^{ - } \hfill \\ {\text{8Al}}_{{2}} {\text{Cl}}_{{7}}^{ - } + {\text{ Al}}_{{2}} {\text{S}}_{{{12}}}^{*} - {\text{FeN}}_{{4}} + {\text{FeN}}_{{4}} + {\text{ 6e}}\mathop{\longrightarrow}\limits^{{}}{\text{2Al}}_{{2}} {\text{S}}_{{6}}^{*} - {\text{FeN}}_{{4}} + {\text{ 14AlCl}}_{{4}}^{ - } \hfill \\ {\text{8Al}}_{{2}} {\text{Cl}}_{{7}}^{ - } + {\text{ Al}}_{{2}} {\text{S}}_{{6}}^{*} - {\text{FeN}}_{{4}} + {\text{ 6e}}\mathop{\longrightarrow}\limits^{{}}{\text{2Al}}_{{2}} {\text{S}}_{{3}} + {\text{ FeN}}_{{4}} + {\text{ 14AlCl}}_{{4}}^{ - } \hfill \\ \end{gathered}$$

The overall reaction on cathode side:

$${\text{8Al}}_{{2}} {\text{Cl}}_{{7}}^{ - } + {\text{ 3S}}_{{}} + {\text{ 6e}}\mathop{\longrightarrow}\limits^{{ FeN_{4} }}{\text{Al}}_{{2}} {\text{S}}_{{3}} + {\text{ 14AlCl}}_{{4}}^{ - }$$

Meanwhile, the reaction on anode side can be written below:

$${\text{Al + }}7{\text{AlCl}}_{4}^{ - } { - }3{\text{e}}\mathop{\longrightarrow}\limits^{{}}4{\text{Al}}_{2} {\text{Cl}}_{7}^{ - }$$

Accordingly, the overall reaction is given below:

$${\text{2Al }} + {\text{ 3S}}\mathop{\longrightarrow}\limits^{{{\text{FeN}}_{4} }}{\text{Al}}_{{2}} {\text{S}}_{{3}}$$

**Fig. 5:**
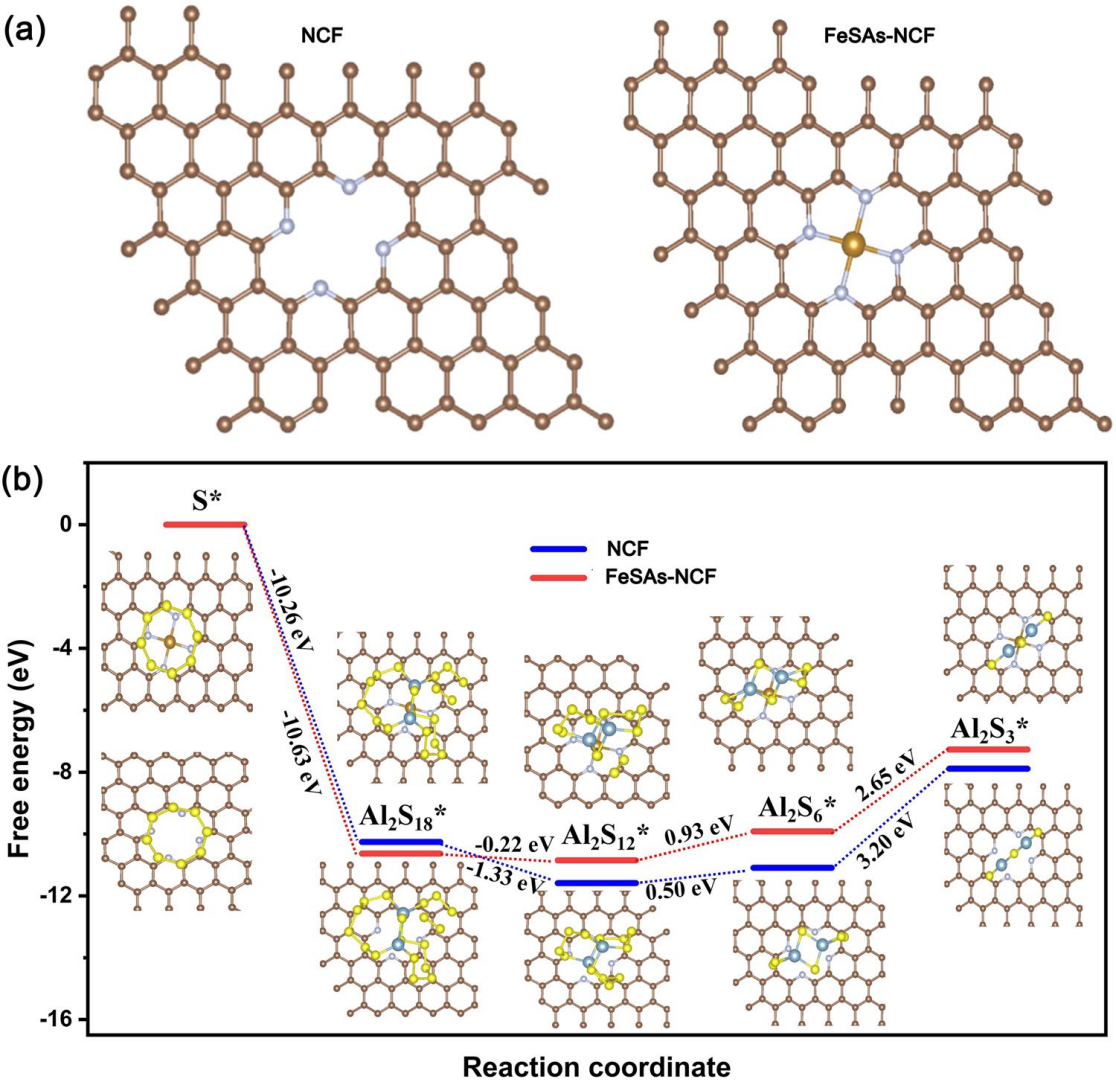
**a** The optimized structure models of NCF and FeSAs-NCF and **b** Energy profiles for the reduction of sulfur to aluminum sulfides on NCF and FeSAs-NCF

The first-principle calculations were performed to further reveal the improved reaction kinetics of the aluminum polysulfides. The optimized structure models of NCF and FeSAs-NCF are displayed in Fig. 5a. The overall reduction from S_8_ to Al_2_S_3_ involved in multiple intermediates including Al_2_S_18_, Al_2_S_12_, and Al_2_S_6_ during the discharge was considered [6, 45]. The optimized adsorption conformations of various polysulfide intermediates on the NCF and FeSAs-NCF substrates are shown in Fig. 5b. The Gibbs free energies were calculated of each reaction on both the NCF and FeSAs-NCF substrates, and the corresponding Gibbs free energy profiles are shown in Fig. 5b. After the spontaneous exothermic process of converting S_8_ to Al_2_S_18_, where the steps of the formation of Al_2_S_12_, Al_2_S_6_ and Al_2_S_3_ are either endothermic or nearly thermoneutral, the largest positive Gibbs free energy of the formation of Al_2_S_3_ from Al_2_S_6_ reveals that this process is the rate-determining step in the entire discharge reaction. The Gibbs free energy on the FeSAs-NCF (2.65 eV) is lower than that of on the NCF (3.20 eV) for the reduction of Al_2_S_6_, demonstrating the reduction of S is thermodynamically more favorable on the FeSAs-NCF than on the NCF substrate.

## Conclusions

In summary, iron single atoms dispersed in nitrogen-doped carbon nanofibers have been successfully prepared and used as a unique interlayer to modify the separator for Al–S batteries. The HAADF-STEM and XANES characterizations confirm the iron is atomically dispersed with the coordination of Fe–N_4_. The Al–S battery with the FeSAs-NCF demonstrates significantly enhanced electrochemical performance, and the Al–S battery with FeSAs-NCF delivers a specific capacity of 780 mAh g^−1^. The enhanced performance is ascribed to the unique spatial configuration and chemical properties of FeSAs-NCF. Specifically, the FeSAs-NCF is functioned as a chemical and physical barrier to hinder the shuttle effect of soluble aluminum polysulfides. More importantly, the Fe single atoms can catalyze the conversion of aluminum polysulfides to promote the kinetics of the charge/discharge processes.

## Supplementary Information

Below is the link to the electronic supplementary material.

Supplementary file1 (PDF 869 kb)
