# Supplementary material for: Atomically Dispersed Iron Active Sites Promoting Reversible Redox Kinetics and Suppressing Shuttle Effect in Aluminum–Sulfur Batteries
Source: Nanomicro Lett. 2022 Aug 20;14:169. doi: 10.1007/s40820-022-00915-4 (PMC9392677; doi:10.1007/s40820-022-00915-4)
Supplement: Supplementary file 1 — Supplementary file1 (PDF 869 kb) [file 40820_2022_915_MOESM1_ESM.pdf]

Supporting Information for

Atomically Dispersed Iron Active Sites Promoting Reversible Redox Kinetics and Suppressing Shuttle Effect in Aluminum-Sulfur Batteries

Fei Wang[1, +], Min Jiang[1, +], Tianshuo Zhao[1], Pengyu Meng[1], Jianmin Ren[1], Zhaohui Yang[1], Jiao Zhang[1], Chaopeng Fu[1, *], Baode Sun[1]

[1]School of Materials Science and Engineering, Shanghai Jiao Tong University, Shanghai 200240, P. R. China

[+]Fei Wang and Min Jiang contributed equally to this work.

*Corresponding author. E-mail: chaopengfu@sjtu.edu.cn (Chaopeng Fu)

S1 Experimental Section

All the samples were handled in an argon-filled glovebox (MBraun) with a recirculation system and water, under a controlled argon atmosphere (H_2O and O_2 < 0.1 ppm). The chemical operations were carried out either on the bench under Ar (99.9999%) using standard Schlenk techniques (with vacuum < 0.1 Pa) or in glovebox.

S1.1 Synthesis of Fe-Phen

$FeCl_2 \cdot 4H_2O$ (0.5 mmol) and 1,10-phenanthroline (3 mmol) were mixed in ethanol (60 mL) and stirred through refluxing under N_2 at 60 °C. The resultant dispersion was then heated at 80 °C for 12 h under vacuum to evaporate the ethanol to yield solid powder.

S1.2 Synthesis of ZnO Nanoparticles

ZnO nanoparticles were synthesized according to the previous work (Small 2017, 13, 1700238). Typically, an aqueous solution (40 mL) containing of $Zn(CH_3COO)_2 \cdot 2H_2O$ (0.88 g, 4.0 mmol) was added to another aqueous solution containing 200 ml H_2O and TEA (triethanolamine, 6.02 g, 20.0 mmol). After vigorous sonication for 30 mins at room temperature, the resulting liquid was aged for 12 h at room temperature. The ZnO nanoparticles were centrifuged (8000 rpm) and washed with water and ethanol several times.

S1.3 Synthesis of the Precursor of FeSAs-CNF

0.60 g of PAN (Mw = 150,000) was added to the 3.5 mL of DMF solution and stirred for 1 h to form a homogeneous solution (denoted as solution A). Meanwhile, the ZnO nanoparticles (0.90 g) were dispersed ultrasonically in 1.5 mL of DMF to form a uniform suspension (denoted as solution B). Then, the solution A and B were mixed. Finally, 0.05 g of Fe-Phen and 0.1g of 1,10-phenanthroline were added into the above solution and further stirred for 12 h at room temperature to form a homogeneous and viscous solution. Subsequently, the precursor solution was loaded into a syringe with a 21# needle and electrospun with a constant flow rate of 0.5 mL h^{-1} at a high voltage of 18 kV. The distance between the needle and the receiver was 16 cm. Aluminum foil was used the collector with the rotating speed of 200 rpm min^{-1}. The precursor of FeSAs-CNF was obtained.

S1.4 Fabrication of Symmetric Cells

Symmetric cells of the S_6^{2-} containing electoryte: For the FeSAs-NCF symmetric cell, the FeSAs-NCF electrode was used as both anode and cathode, and the electrolyte was ionic liquid containing S_6^{2-}. For the NCF symmetric cell, the NCF electrode was used as both anode and cathode, and the electrolyte was ionic liquid containing S_6^{2-}.

Symmetric cells of FeSAs catalyzing Al_2S_3: 40 mg of Al_2S_3 was mixed with 40 mg of FeSAs-NCF, 10 mg of carbon black and 10 mg of polyvinylidene fluoride (PVDF) in methylpyrrolidone (NMP). The FeSAs-NCF/Al_2S_3 slurry was casted onto the current collector for drying at 60 °C for 24 h. The Al_2S_3/NCF electrode were obtained with the identical method. For the FeSAs-NCF/Al_2S_3 symmetric cell, the FeSAs-NCF/Al_2S_3 electrode was used as both anode and cathode, and the electrolyte was [EMIM]Cl/$AlCl_3$ ionic liquid. For the NCF/Al_2S_3 symmetric cell, the NCF/Al_2S_3 electrode was used as both anode and cathode, and the electrolyte was [EMIM]Cl/$AlCl_3$ ionic liquid.

S2 Supplementary Figures and Tables

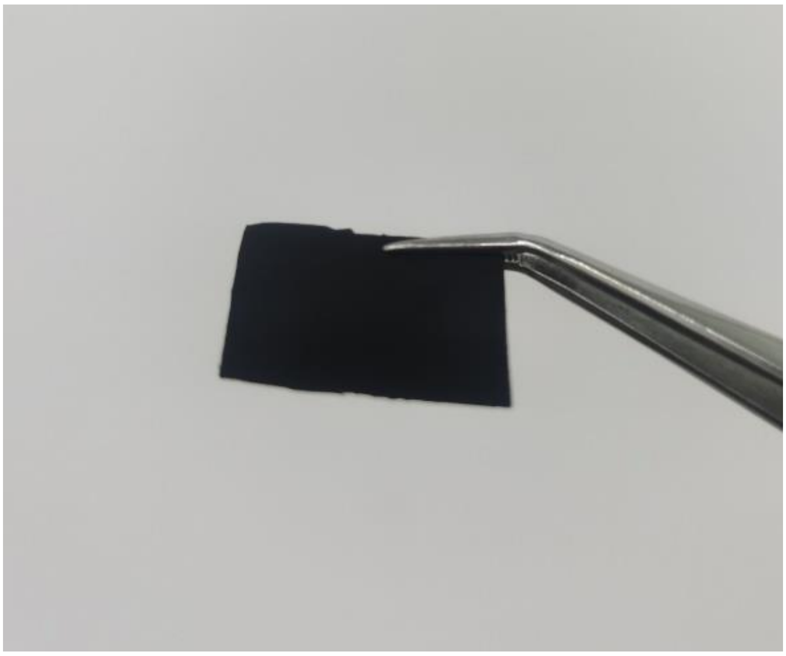

Fig. S1 A photograph of the FeSAs-NCF sheet

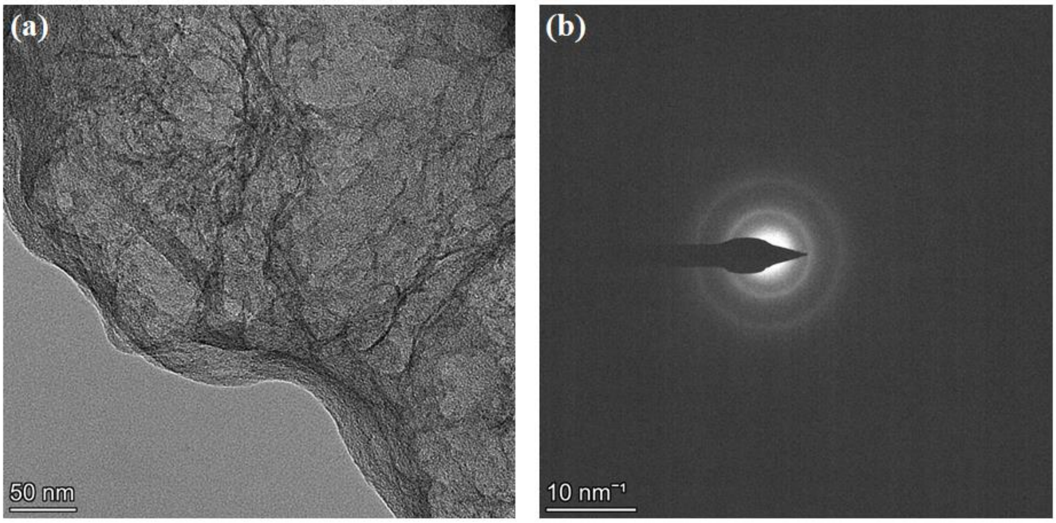

Fig. S2 a TEM image and **b** the corresponding selected area electron diffraction (SAED) pattern of the FeSAs-NCF

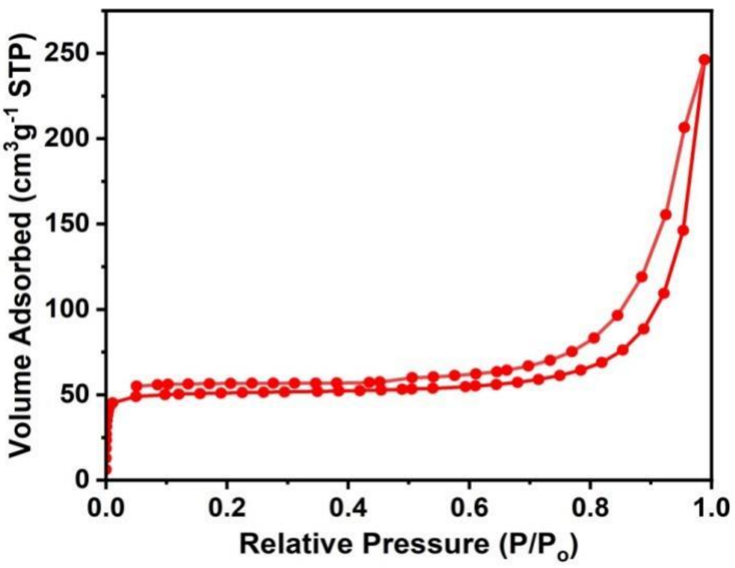

Fig. S3 Nitrogen adsorption-desorption isotherm of the FeSAs-NCF

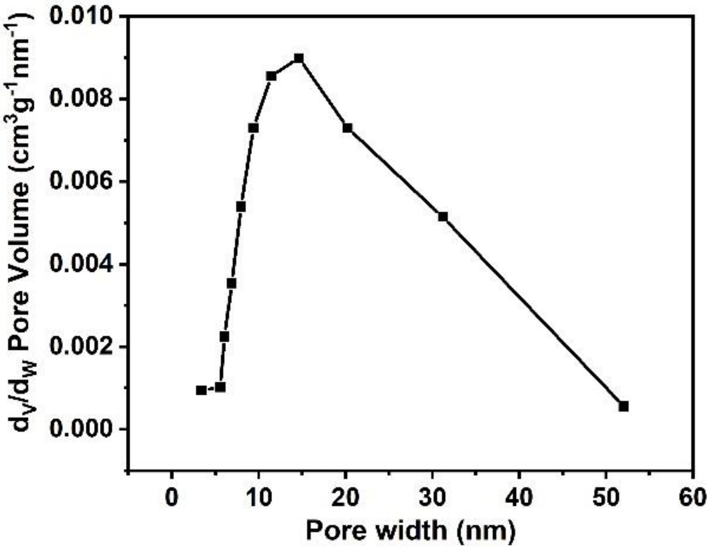

Fig. S4 Pore size distribution of the FeSAs-NCF

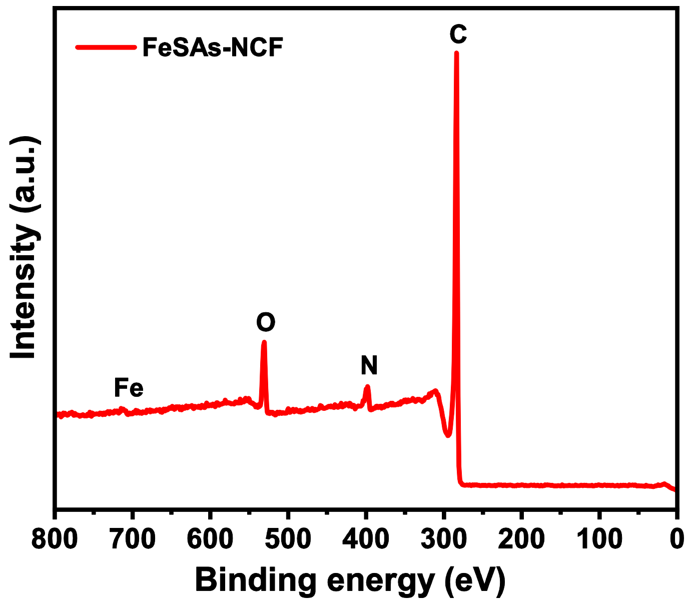

Fig. S5 XPS survey scan of the FeSAs-NCF

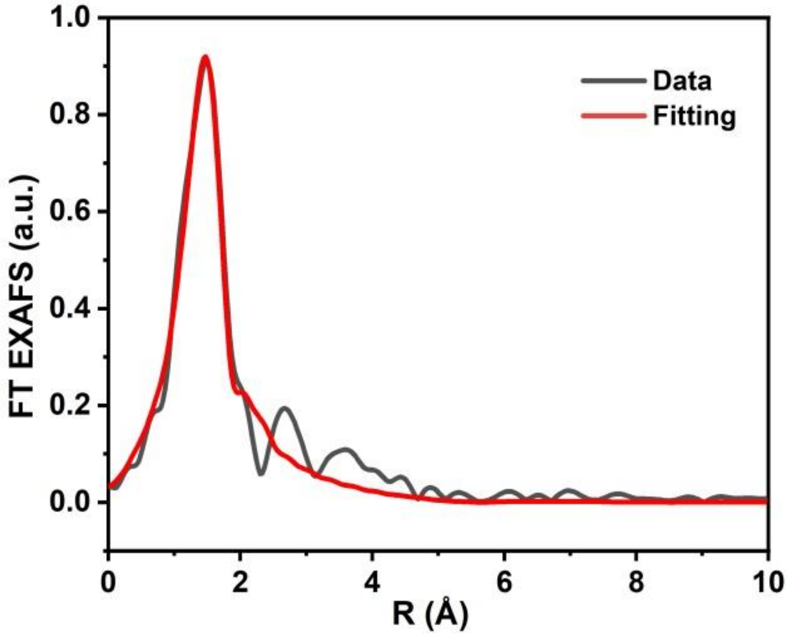

Fig. S6 The fitting Fourier transformed (FT) k^2-weighted-EXAFS spectrum of FeSAs-NCF

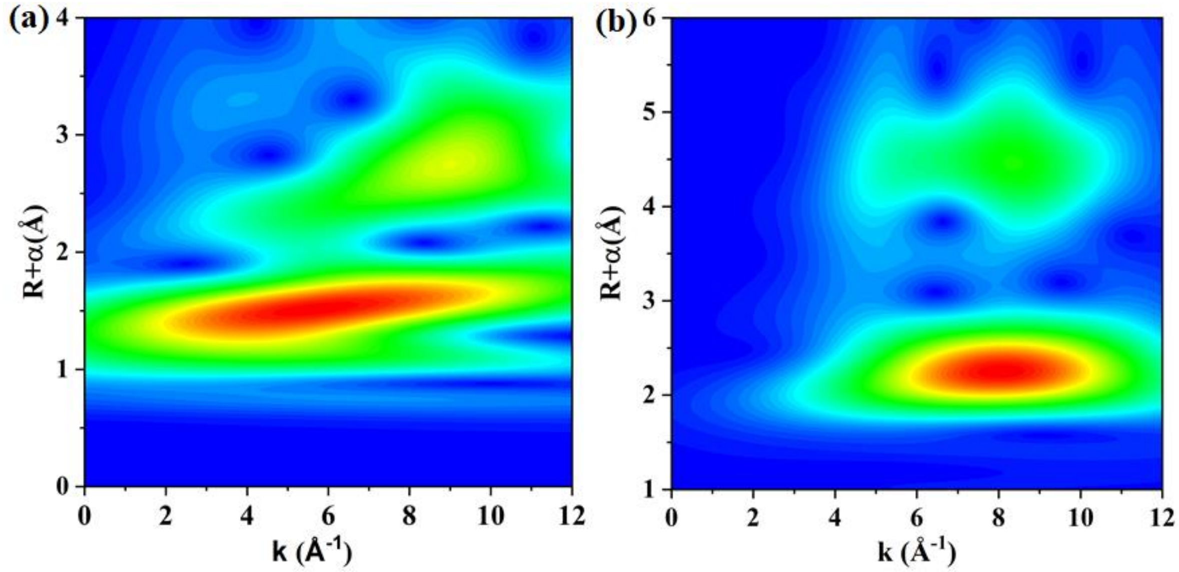

Fig. S7 Wavelet transforms of **a** FePc and **b** Fe foil

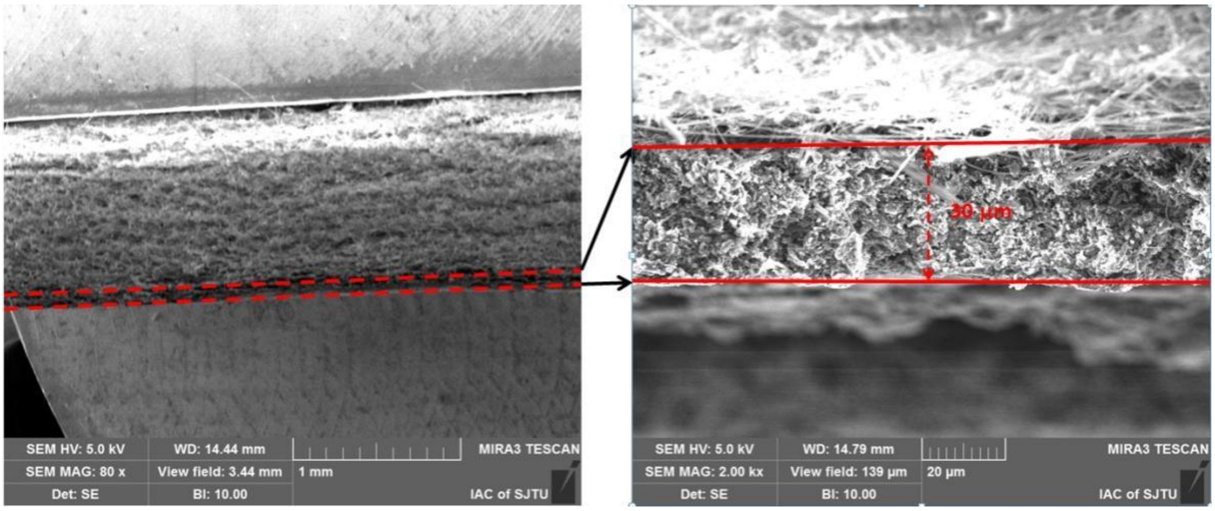

Fig. S8 The cross-section SEM images of the FeSAs-NCF modified separator

Then, the diffusion coefficients were calculated from the Nyquist plots in Fig. 3b through the following equations (1) and (2):

$$D = \frac{1}{2}\left[\frac{RT}{F^2n^2AC\sigma}\right]^2 \qquad (S1)$$

$$Z_{R_e} = K + \sigma\omega^{-1/2} \qquad (S2)$$

R is the gas constant is 8.314 J K^{-1} mol^{-1}; T is the absolute temperature of 298 K; F is the Faraday constant of 96485 C mol^{-1}; n is the number of transferred electrons; A is the active surface area; C is the concentration of $Al_2Cl_7^-$ ions in the cathode electrode (~ 8.64 $\times 10^{-3}$ mol cm^{-3}); σ is the Warburg coefficient. Thus, the diffusion coefficients of cells with FeSAs-NCF, NCF and blank separators are 9.63×10^{-15}, 2.70×10^{-15} and 7.43×10^{-16} cm^2 s^{-1}.

Meanwhile, the ion diffusion coefficient of the cell with FeSAs-NCF was also studied to confirm the accelerated reaction kinetic and evaluated through the galvanostatic intermittent titration technique (GITT) as follow.

The diffusion coefficient of aluminum-ion can be qualitatively evaluated by Eqs. (S3) and (S4):

$$D = \frac{4}{\pi t}\left(\frac{n_m V_m}{S}\right)^2\left(\frac{\Delta E_s}{\Delta E_t}\right)^2 \qquad (S3)$$

$$V_m = \frac{V_c N_a}{N} \qquad (S4)$$

where t refers to the relaxation time, n_m and V_m are the molar number and molar volume of the active material, S represents the surface area of the cathode, ΔE_s and ΔE_t are the voltage change triggered by the pulse and galvanostatic charge, V_c is the cell volume of the sulfur, N refers to the number of molecules in the cell, N_a represents Avogadro's number. The results display that the ratio of D_1 (the cell with FeSAs-NCF) to D_2 (the blank cell) is greater than 1, revealing that the FeSAs-NCF can accelerate the ion diffusion to contribute to fast reaction kinetic.

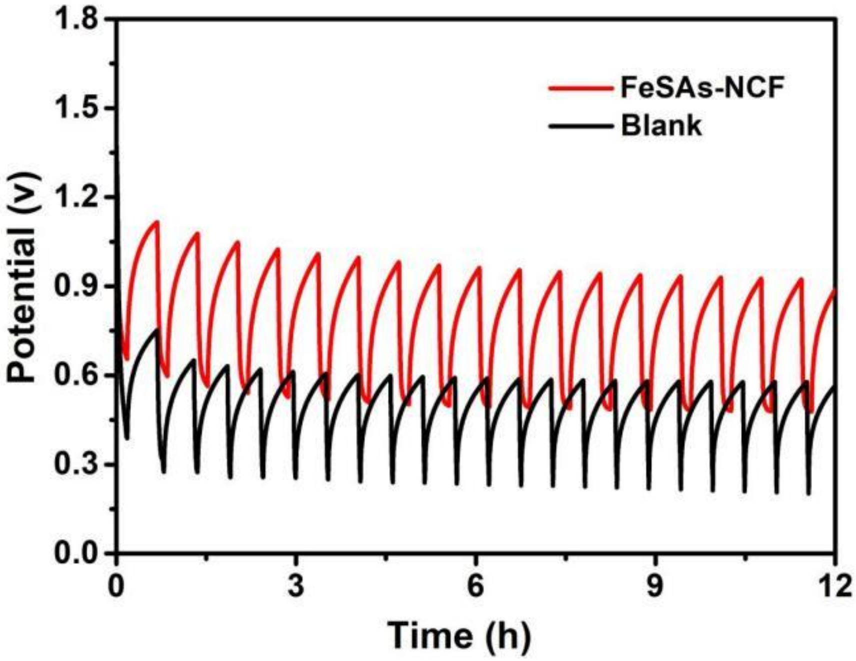

Fig. S9 GITT curves of the cells with FeSAs-NCF modified separator and blank separator during the charge and discharge process

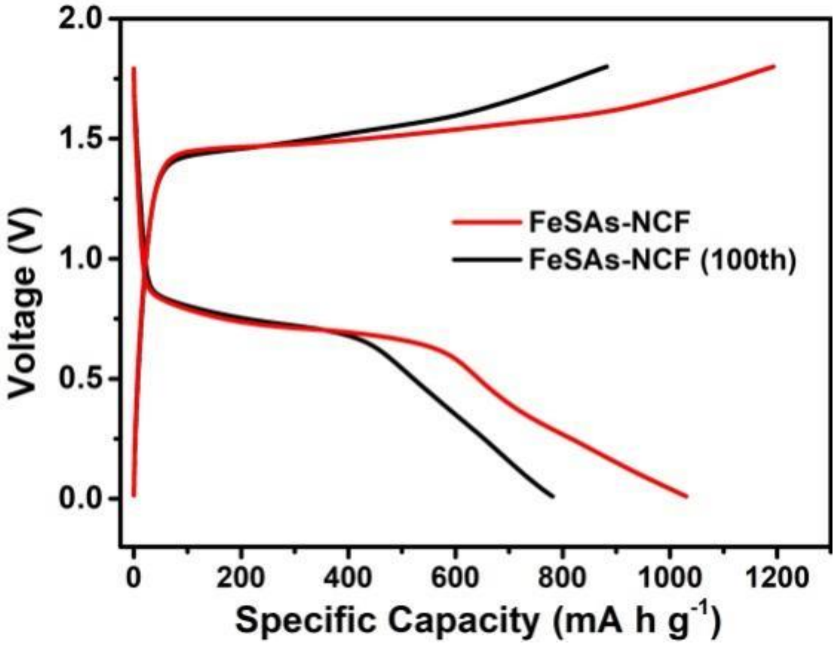

Fig. S10 Charge-discharge curves of the Al-S batteries with FeSAs-NCF at second and 100th cycles

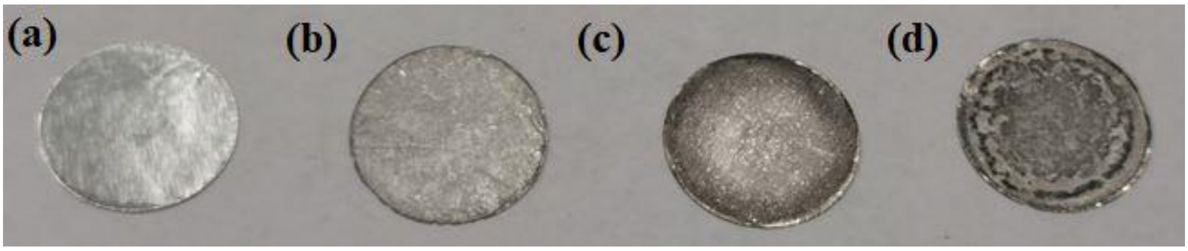

Fig. S11 The photographs of (**a**) the pristine Al foil, Al foils in the batteries with (**b**) FeSAs-NCF, (**c**) NCF, and (**d**) blank separators at a current density of 100 mA g^{-1} after 100 cycles

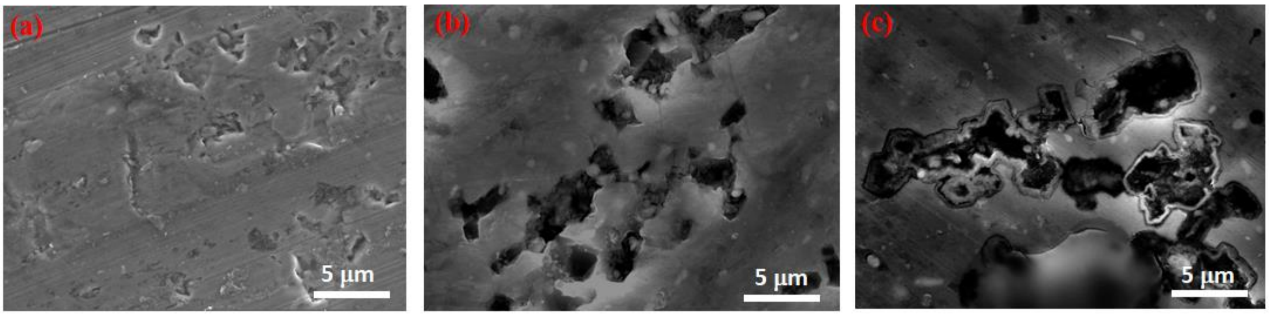

Fig. S12 SEM images of Al anodes in the Al-S batteries with (**a**) FeSAs-NCF (**b**) NCF and (**c**) blank separators at a current density of 100 mA g^{-1} after 100 cycles

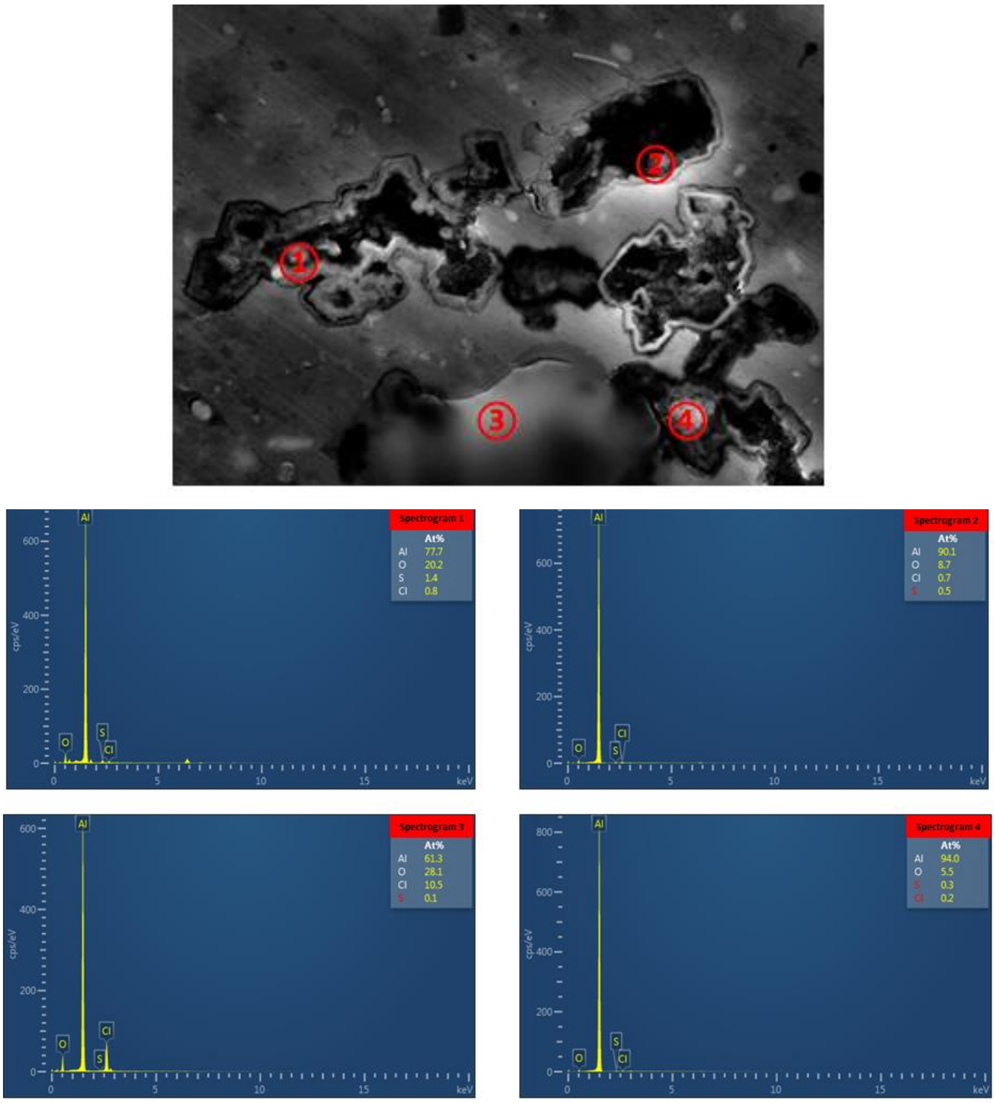

Fig. S13 The EDS mappings of the Al anode from the blank cell after 100 cycles

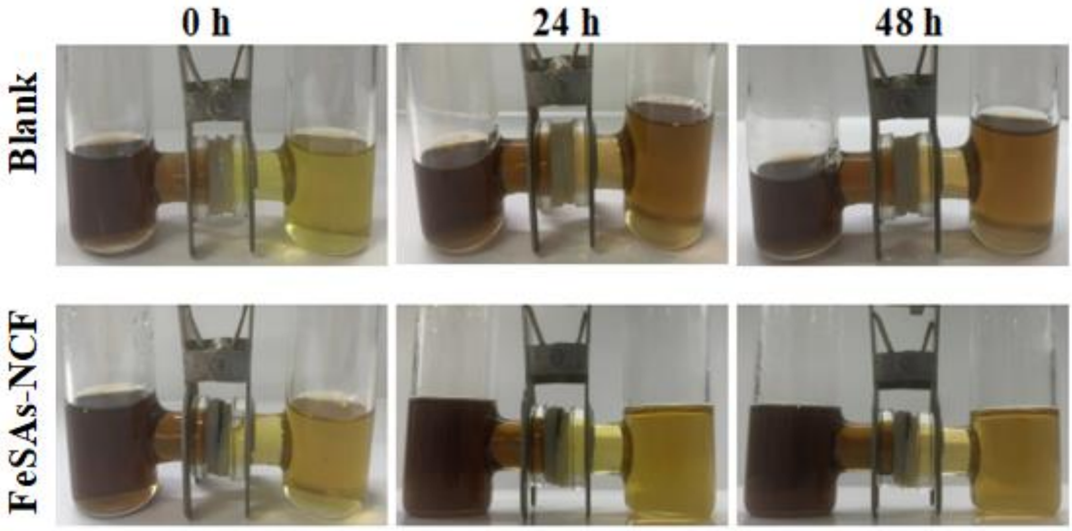

Fig. S14 The polysulfides permeation tests for blank separator and FeSAs-NCF modified separator

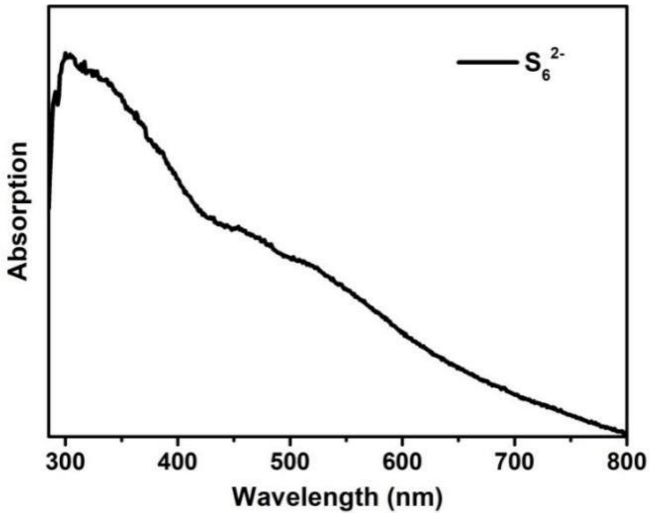

Figure S15 UV-vis spectrum of the electrolyte containing aluminum polysulfide (S_6^{2-})

Table S1 The fitted values of Fe K-edge EXAFS of the Fe SAs-NCF

**Sample**	**Edge**	**Path**	**N**	**R(Å)**	**σ^2(Å^2)**	**ΔE_0**	***R*-factor**
**FeSAs-NCF**	Fe	Fe-N	3.52	1.97	0.01166	-3.95	0.028

Table S2 The fitted R_{ct} values of the Al-S batteries with Fe SAs-NCF, NCF and blank sample

**Sample**	FeSAs-NCF	NCF	blank
**R_{ct} (Ω)**	553	915	1112

Table S3 Comparison of the Al-S battery performances with others reported in recent literature

**Material system**	**Initial discharge capacity / mAh g^{-1}**	**Discharge capacity mAh g^{-1}**	**Cycles**	**Refs.**
Ketjen black carbon/S	1200@120 mA g^{-1}	~50@30 mA g^{-1}	4	J. Power Sources, 2015, 283, 416–422
Activated carbon cloth/S	1320@50 mA g^{-1}	1000@50 mA g^{-1}	20	Angew. Chem. Int. Ed., 2016, 55, 9898–9901
S@CMK-3	1390@251 mA g^{-1}	~400@251 mA g^{-1}	20	Angew. Chem. Int. Ed., 2018, 57, 1898–1902

Carbonised-ZIF/S	1410@200 mA g^{-1}	420@200 mA g^{-1}	30	Chem. Commun., 2020, 56, 2023–2026
S@HKUST-1-C	1200@1000 mA g^{-1}	460@1000 mA g^{-1}	500	Adv. Funct. Mater., 2019, 29, 1807676
CNF/S	~1200@0.05C	450@0.05C	10	Adv. Energy Mater., 2017, 7, 1700561
SWCNT/S	~900@100 mA g^{-1}	~300@100 mA g^{-1}	30	ACS Appl. Energy Mater., 2020, 3, 6805–6814
CNF/S as cathode	1000@0.05C	600@0.05C	50	Chem, 2018, 4, 586–598
Graphene/CoS_2/S	1145@50 mA g^{-1}	680@50 mA g^{-1}	37	Sustainable Energy Fuels, 2020, 4, 1630–1641
TiN@N-doped-graphene/S	~993@100 mA g^{-1}	500@100 mA g^{-1}	200	*Energy Storage Mater.*, 2022, *48*, 297-305.
S@CoNG	~1026@50 mA g^{-1}	820@50 mA g^{-1}	300	Angew. Chem. Int. Ed., 2022, e202202696
CoNG/S	1631@200 mA g^{-1}	~500@200 mA g^{-1}	10	*J. of Energy Chem.*, 2022, *67*, 354-360.
S@$Ti_3C_2T_x$	489@300 mA g^{-1}	415@300 mA g^{-1}	280	*Science China Mate.*, 2022, 1-13.
**FeSAs-NCF**	**1130@100 mA g^{-1}**<br>**875@1000 mA g^{-1}**	**780@100 mA g^{-1}**<br>**320@1000 mA g^{-1}**	**100**<br>**500**	**This work**
